# Distinguished Functions of Microglia in the Two Stages of Oxygen-Induced Retinopathy: A Novel Target in the Treatment of Ischemic Retinopathy

**DOI:** 10.3390/life12101676

**Published:** 2022-10-21

**Authors:** Ziyi Zhou, Yutong Jing, Yali Niu, Tianfang Chang, Jiaxing Sun, Changmei Guo, Yusheng Wang, Guorui Dou

**Affiliations:** Department of Ophthalmology, Eye Institute of Chinese PLA, Xijing Hospital, Fourth Military Medical University, Xi’an 710032, China

**Keywords:** microglia, PLX5622, ischemic retinopathy, angiogenesis, vessel remodeling

## Abstract

Microglia is the resident immune cell in the retina, playing the role of immune surveillance in a traditional concept. With the heated focus on the mechanisms of microglia in pathological conditions, more and more functions of microglia have been discovered. Although the regulating role of microglia has been explored in ischemic retinopathy, little is known about its mechanisms in the different stages of the pathological process. Here, we removed microglia in the oxygen-induced retinopathy model by PLX5622 and revealed that the removal of activated microglia reduced pathological angiogenesis in the early stage after ischemic insult and alleviated the over-apoptosis of photoreceptors in the vessel remodeling phase. Our results indicated that microglia might play distinguished functions in the angiogenic and remodeling stages, and that the inhibition of microglia might be a promising target in the future treatment of ischemic retinopathy.

## 1. Introduction

The maintenance of visual acuity requires close coupling between the energy demand of neurons and vascular supply [1]. Retinal vascular supply by the subtle network is formed through sprouting during angiogenesis. They then undergo vascular remodeling, which is characterized by precise vascular pruning, to construct the mature vasculature [2]. Excessive vascular pruning is accompanied by the destruction of barrier function and the damage of neuron functions [3]. Many diseases are caused by disturbed microvascular remodeling and abnormal vessel recovery, such as several ischemic retinopathies including proliferative diabetic retinopathy (PDR) and retinopathy of prematurity (ROP), which are induced by ischemic insult [4,5]. These diseases involve initial microvascular degeneration and a disruptive vascular network, accompanied by pathological neovascularization and insufficient vascular remodeling, which finally leads to neuroretinal damage and irreversible blindness [5]. In this perspective, exploring the underlying mechanisms in regulating neovascularization and vessel remodeling suggests a better option for the treatment of ischemic retinopathy.

The microglia are a type of specialized immune cell located in the central nervous system (CNS) [6], which was traditionally considered merely as innate immune cells [7]. Notably, an increasing number of studies have confirmed that microglia act not only as immune cells to interfere with external stimuli but also participate in the normal vascular development of the retina [8], affecting the early development and remodeling of the retinal vasculature [9]. Microglia contact the endothelial tip cell filopodia and guide vascular growth in the formation of retinal vasculature. The consumption of retinal microglia leads to delayed retinal vascular development [10]. It is particularly worth mentioning that in the ischemic retina, microglia accumulated in the ischemia and NV regions and were considered to be the driving force of the pathological process [11,12]. After being activated under the circumstance of ischemic insult, microglia secreted proinflammatory and anti-inflammatory cytokines and reversed the cellular microenvironment by clearing neurons and exclusive vessel units [13,14]. Recently, Denes et al. [15] revealed that microglia established direct connections with different neuronal compartments, which could regulate neuronal apoptosis. However, the role of this immune cell in the formation and regression of neovascular drafts has not been fully illustrated.

The retina is a part of the central nervous system; thus, the limited regenerative capacity of the retina is indicated [1]. A relative exception to the feature is retinal vessels, which have a greater tendency to remodel in order to meet the metabolic demands. There are a number of researches on the role of microglia in vessel formation [16,17,18]. We focused on the oxygen-induced retinopathy (OIR) model, which was characterized by separate stages of vascular degeneration, neovascularization, and regression [19]. Microglia promoted angiogenesis after vascular occlusion in OIR, thus producing less NV through VEGF and bFGF in the first stage of the OIR model [20]. Meanwhile, little is known about the role it played in the stage of vessel remodeling and the regression of the neovascular clusters. Here, we explored the specific role of microglia in the two stages of OIR. We used PLX5622, an effective and specific oral bioavailability and brain permeability colony-stimulating factor 1 receptor (CSF1R) inhibitor [21], which specifically targeted the development, maintenance, and activation of microglia, to consume microglia in mice until pathological vascular reshaping was almost completed. PLX5622 is widely used in the study of microglia in the central nervous system, for it can specifically and efficiently eliminate microglia [21]. With the elimination of microglia, we found that microglia played vital roles in both the formation and regression of pathological vascular clusters, photoreceptor apoptosis, and visual injuries in the OIR mice. Moreover, these results revealed that microglia had distinct roles in different stages of the disease and presented the possibility of inhibiting the over-activation of immune cells in the future treatment of ocular neovascular diseases.

## 2. Materials and Methods

### 2.1. Mice

The CX3CR1-GFP mice were maintained in our laboratory. The oxygen-induced retinopathy (OIR) model was constructed as described [22]. On postnatal day 7 (P7), we placed the pups and their feeding mothers into a high-oxygen box (with an oxygen concentration of 75 ± 2%) for 5 days and took them back to room air on P12. Samples were collected at P12 or P17.

All animal experiments were approved by the Animal Experiment Administration Committee of the Fourth Military Medical University (Xi’an, China). All the ethnical and humane treatment of animals were ensured. We strictly observed the guideline in the National Institutes of Health Guide for the Care and Use of Laboratory Animals prepared by the National Academy of Science as the study was carried out.

### 2.2. PLX5622 Administration

CSF1R inhibitor PLX5622 (Selleck, China) was given to the CX3CR1-GFP mice. From P12, when the mice were taken back to room air, the natal mice were treated daily with oral gavage with 100 μL of solution (10 mg/mL) in CMC-Na (Selleck, China) per 10 g of body weight (at the dose of 100 mg/kg body weight).

### 2.3. Weight Monitor

After the mice were removed from the oxygen box, each pup was weighed simultaneously every day with a fine scale. This was repeated three times to take the average value and calculate the growth rate according to the initial weight of each pup.

### 2.4. Flow Cytometry

Collagenase I (1 mg/mL) and DNase I (1 mg/mL) were used to digest retinal cells from CX3CR1-GFP mice for 40 min. Cell screens were used to filter out excess impurities. Samples were resuspended with FACs buffer and were analyzed with a FC500 flow cytometer (BeckmanQ38). Samples from WT were used for negative control to define the cell population. The antibody used are listed in Table 1. FlowJo 7.6 software was used to analyze the data.

### 2.5. Staining

Sections of HE were cut at a thickness of 4 µm and stained according to routine protocols [23]. The slices near the optic nerve were selected. Slideviewer was used to measure the thickness of each layer of the retina according to the magnification.

For immunofluorescence staining, retina samples were dissected and pre-fixed in 4% paraformaldehyde (PFA) overnight at 4 °C. Then, the samples were blocked and permeabilized in PBS containing 1% bovine serum albumin (BSA) and 1% Triton X-100 overnight. We incubated samples with anti-CD31 (1:400) or αSMA (1:200) in PBS containing 1% BSA and 1% Triton X-100 overnight at 4 °C, followed by incubation with Alexa Fluor^®^594 goat anti-rabbit immunoglobulin G (IgG; H + L) secondary antibody (1:100) and FITC donkey anti-rat immunoglobulin G (IgG; H + L) secondary antibody (1:100) in PBS. DAPI staining was performed according to the manufacturer’s instructions. We washed the samples three times for 5 min between each step. Photographs were taken by a confocal laser scanning microscope (FV1000, Olympus). The antibodies mentioned are listed in Table 1. The images were reconstructed by Imaris 9.5 version.

### 2.6. RT-PCR

All the samples were extracted by TRIzol and reverse-transcribed into cDNA by the PrimeScrip RT reagent kit (TaKaRa Biotechnology, Dalian, China) according to the instructions. The SYBR premix ExTaqII (TaKaRa Biotechnology) and Biosystems 7500 Real-time PCR system (Applied Biosystems, Foster City, CA, USA) were used to perform quantitative real-time PCR (qPCR). The 2–ΔΔCT method was used to calculate the relative expressions. Primers are listed in Table 2.

### 2.7. ERG Examination

ERG examination was performed on the control and PLX groups of OIR mice on P17 and P25 to evaluate retinal function. Before ERG examination, mice were dark-adapted overnight, anesthetized by an intraperitoneal injection of 0.8% sodium pentobarbital, and treated with mydriatic with 1% tropicamide. The guide electrode was lightly placed onto the cornea, the reference electrode was placed subcutaneously behind the ear, and the ground electrode was connected to the base of the tail. Exposed to the program of dark adaptation, light adaptation, and light adaptation flicker, the responses of both eyes were recorded. The results showed in the article were the section of MAX and OPS in ERG. The MAX wave represented the overall function of the retina and OPS wave represented the vascular function. The average value of the a and b wave amplitudes in the MAX wave and the O2 wave in the OPS wave were used for statistical analysis.

### 2.8. TUNEL Assay

Retinal sections were stained with TUNEL using an apoptosis in situ detection kit. A total of 30 μm retinal cryosections were air-dried and washed with phosphate-buffered saline (PBS) for 5 min, and the process was repeated 3 times. Then, the samples were penetrated in the PBS with 0.2% TritonX-100 for 5 min. Equilibration solution was used to balance the pH for 10 min. Then, equilibration buffer containing nucleotides and the rTdT enzyme was used to stain at 37 °C for 1 h in the dark. The 2x SSC buffer was added to stop the reaction, and the samples were observed and photographed under an ultra-high-resolution laser confocal microscope. The slices near the optic nerve were selected.

### 2.9. Statistics

Statistical analysis was performed with the Image-Pro Plus 6.0 and GraphPad Prism 5 software. The analysis of IF results were performed by Image-Pro Plus 6.0. All quantitative data were presented as mean ± SEM. Statistical significance was calculated using unpaired or paired Student’s *t*-test. *p* < 0.05 was considered significant.

## 3. Results

### 3.1. Plx5622 Effectively Removed Retinal Microglia without Affecting the Whole Conditions of Mice

**Figure 1:**
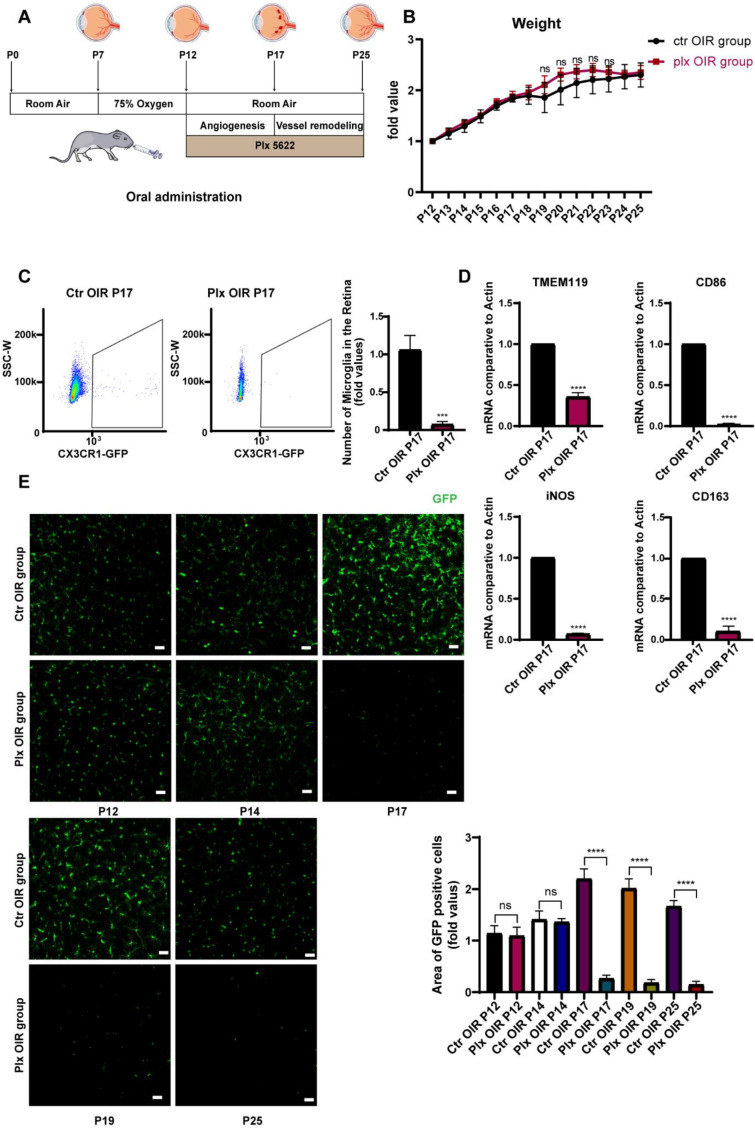
PLX5622 administration effectively removed microglia in the retina. (**A**) Natal mice were placed in an oxygen box with 75% (±2%) from P7 to P12 to establish the OIR model, then were switched to room air at P12. They were subjected to oral administration of PLX5622 from P12 to P25. (**B**) The weights of mice in the two groups were tested each day (n = 3 per group). (**C**) Retina samples were collected from the OIR and Plx groups on P17. The expression of CX3CR1 (green, GFP) on MG was analyzed by FACs (n = 3 per group). (**D**) mRNA expression of microglia (TMEM119), M1 subtype-related genes (iNOS, CD86), and M2 subtype-related genes (CD163) were determined by qRT-PCR (n = 3 per group). (**E**) Immunofluorescence staining of CX3CR1 (GFP, green) in the retinas of CX3CR1-GFP mice were collected on P12, 14, 17, 19, and 25 (n = 3 per group). Scale bar, 100 µm. Bars = means ± SD; *** *p* < 0.001; **** *p* < 0.0001; NS, no significance.

Previously, PLX5622 was used to remove microglia mainly by being added to the forage for daily feeding [21,24,25]. It was not feasible for the neonatal mice, as immature mice mostly relied on their breeding mother for lactation. In this study, we tried to remove the retinal microglia in the OIR retina by intragastric administration of PLX5622 for the first time. We put the mice of postnatal day 7 (P7) into an oxygen box with the oxygen concentration maintained at 75 ± 2%. On P12, the mice were taken out and placed in normoxic conditions for the intragastric administration of PLX5622, according to body weight, successively until P25 (Figure 1A). The weights of mice were detected every day. We found that the growth in their weights was not significantly disturbed during continuous administration, compared with that of the control group, implying that PLX5622 systemic administration was not fatal to neonatal mice and had no significant effect on the whole condition of mice (Figure 1B). It has been previously reported that the PLX5622 diet could remove about 97% of microglia by the seventh day [26,27]. After 5 days of continuous administration, i.e., on P17, the efficiency of microglial removal was detected by flow cytometry. It was found that the number of microglia in the PLX group decreased significantly, and the depletion efficiency reached 83.4% (Figure 1C). In addition, RT-qPCR was used to detect the mRNA level of Tmem119, the marker of microglia, and the other markers of the M1 subtype and M2 subtype. Significant decreases were found in the expression of all these markers, indicating that PLX5622 had no selectivity in the clearance of different microglial subtypes (Figure 1D). At the same time, immunofluorescence staining was also applied to determine the clearance efficiency of microglia. During continuous administration of PLX5622, the number of microglia (green, GFP) in the mice retina declined significantly from P17, and the effect could be maintained until P25 (Figure 1E). The vascular changes in the two stages are shown in Figure S1. Other retinal cells were paid attention to on P17 as well (Figure S2). We noticed that the gliosis of macroglia was moderately increased, which might be caused by the loss of macroglia–microglia interaction. We did not see significant morphological and quantitative changes in RGC and the outer segment of photoreceptors. It indicated that PLX5622 had few side effects on other retinal cells besides microglia.

### 3.2. Elimination of Microglia Suppressed the Pathological Process in the Neovascularization Stage of OIR

**Figure 2:**
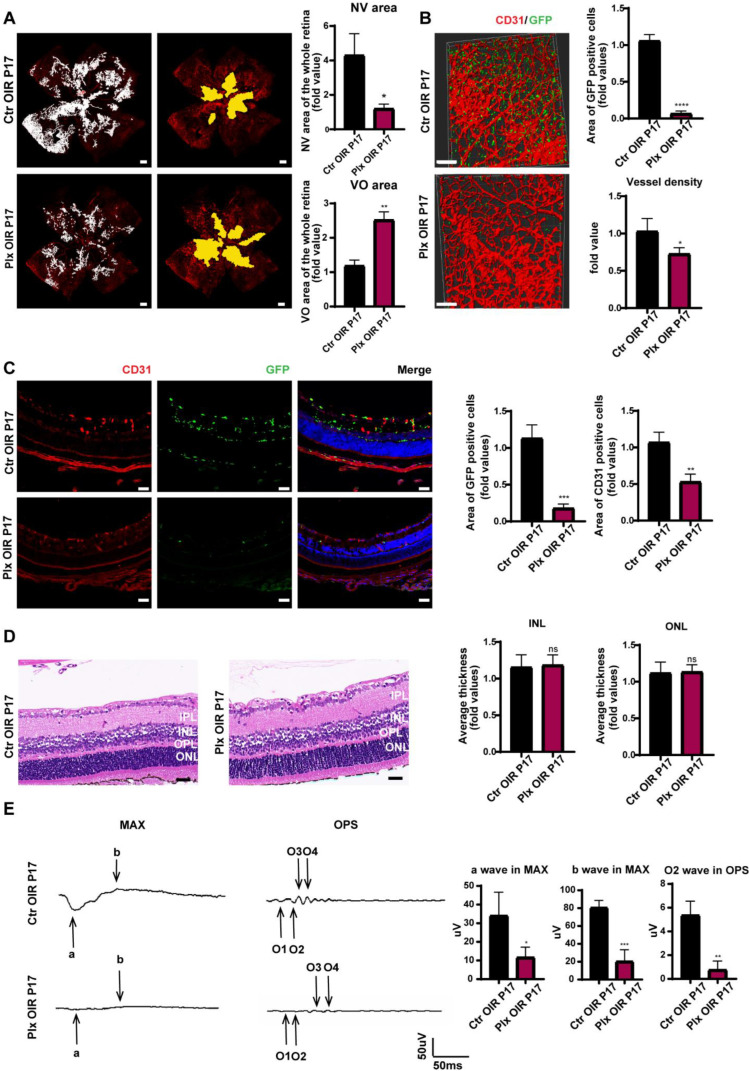
Microglia depletion on P17. (**A**) The whole retinas of CX3CR1-GFP mice were collected on P17, and CD31 (endothelial cells) was stained with red. The white region refers to the NV area and the yellow region refers to the VO area (n = 3 per group) Scale bar, 200 µm. (**B**) CD31 was stained with red. Scale bar, 100 µm. (**C**) Immunofluorescence staining of CD31 (red) and CX3CR1 (GFP, green) in the retinas of CX3CR1-GFP mice were collected on P17 and were reconstructed by Imaris (n = 3 per group). Scale bar, 100 µm. (**D**) HE staining of retinas in the Ctr OIR and Plx OIR groups on P17 (n = 3 per group). Scale bar, 100 µm. (**E**) MAX wave and OPS wave in ERG were tested on P17 (n = 3 per group). Bars = means ± SD; * *p* < 0.05; ** *p* < 0.01; *** *p* < 0.001; **** *p* < 0.0001; NS, no significance.

Next, in order to explore the role of microglia in the two stages of OIR, we paid attention to retinal angiogenesis and visual function after removing microglia. On P17, immunofluorescence staining was used to evaluate retinal vessels. It was found that after the removal of microglia, the area of retinal neovascularization decreased, and the size of the avascular area increased, suggesting that microglia played an essential role in promoting both physiological and pathological neovascularization (Figure 2A). We performed a scan on the longitudinal section of the retina and three-dimensional reconstruction of a retinal vascular plexus. It was observed that the number of neovascular tufts declined significantly after microglial removal (Figure 2B,C). We also found that, after continuous intragastric administration, by the fifth day, we could also effectively remove the vast majority of microglia, with no significant structure changes in the retina (Figure 2D). Shown by the MAX wave in the ERG examination of the mice’s retina, visual function of mice became significantly worse after the removal of microglia at the neovascular stage (Figure 2E). Considering that the structure of the retina in the PLX group was barely different from that in Ctr group, the OPS wave in ERG was conducted, and the wave in the PLX group was much lower (Figure 2E). As it represented the function of retinal vasculature, we proposed that the downgrade of the retinal function was caused by a decreased number of retinal vessels and insufficient retinal blood supply.

### 3.3. Elimination of Microglia Promoted the Repairing Process in Vascular Remodeling Stage of OIR

**Figure 3:**
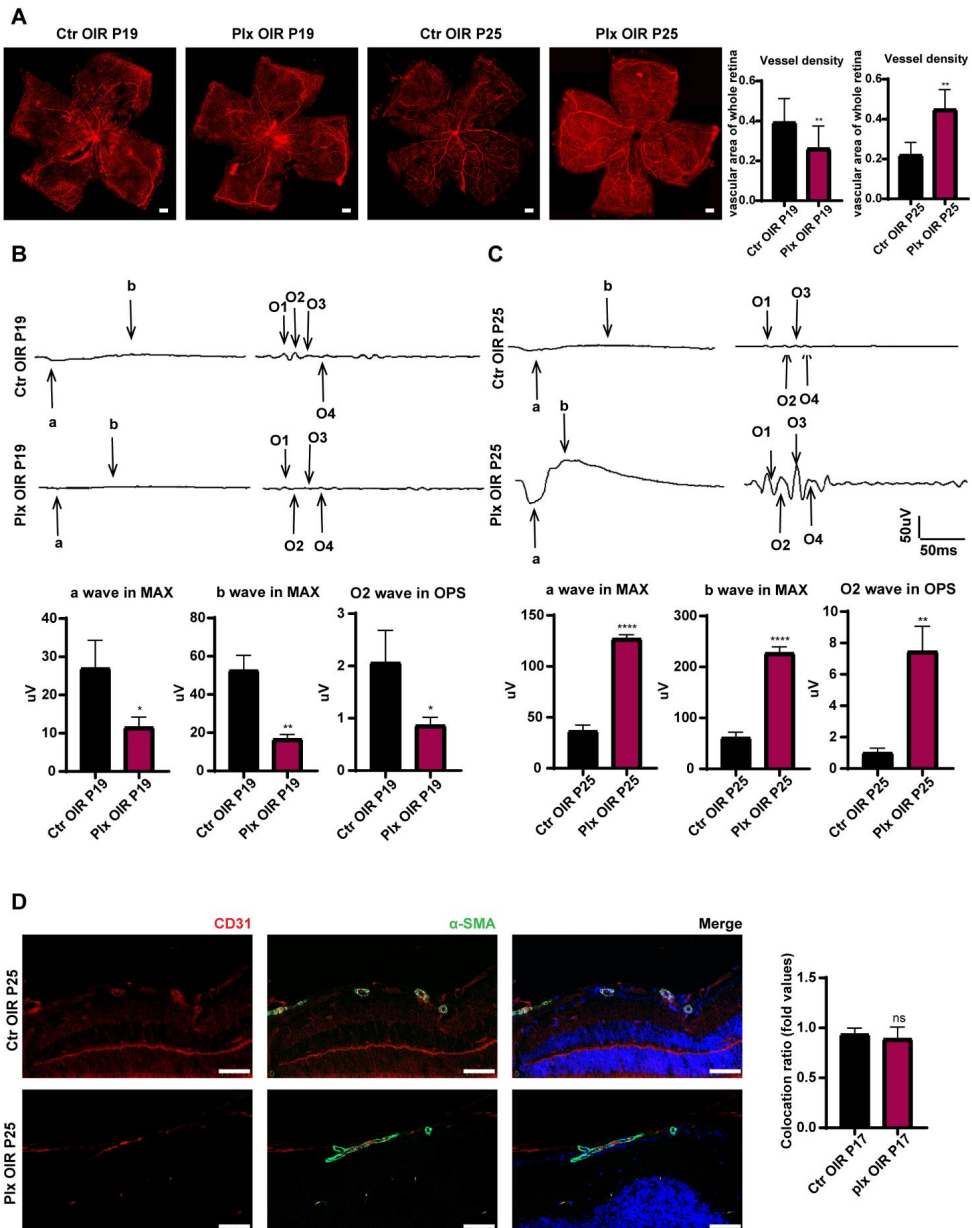
Microglia depletion on P25. (**A**) The whole retinas of CX3CR1-GFP mice were collected on P19 and P25. CD31 was stained with red (n = 3 per group). Scale bar, 200 µm. MAX wave and OPS wave in ERG were tested on P19 (**B**) and P25 (**C**). (**D**) Immunofluorescence staining of CD31 (red), α-SMA (green), and DAPI (blue) on P25 in both Ctr OIR and PLX OIR groups. Scale bar, 100 µm (n = 3 per group). Bars = means ± SD; * *p* < 0.05; ** *p* < 0.01; **** *p* < 0.0001; NS, no significance.

It has been commonly acknowledged that OIR mice were in a vascular remodeling stage from P19 to P25 [28,29]. Interestingly, in the second stage of OIR of vascular remodeling, the depletion of microglia positively impacted the spontaneous regression of pathological clusters. By immunofluorescence staining, we found the vascular density of the PLX group was lower than the vascular density of the Ctr OIR group on P19. However, on P25, the PLX group showed higher vascular density (Figure 3A). The visual function of PLX mice was significantly worse than that of mice in the Ctr OIR group on P19 at the early stage of vascular remodeling. The trend was similar to that on P17. However, when the vascular remodeling was almost completed, the visual function of the PLX group was much better than that of the control group (Figure 3B,C). The pericyte around the vessels took part in the maturation of the vasculature [30]. By immunofluorescence staining of the pericyte by α-SMA, we found that the coverage of vessels by perivascular pericytes presented no significant difference between the two groups (Figure 3D), implying that microglia did not regulate the process of vascular remodeling by regulating the function of perivascular pericytes and the maturation of endothelial cells.

### 3.4. The Expression Profile of Cytokines in the Retina Declined Consistently after Microglia Removal

**Figure 4:**
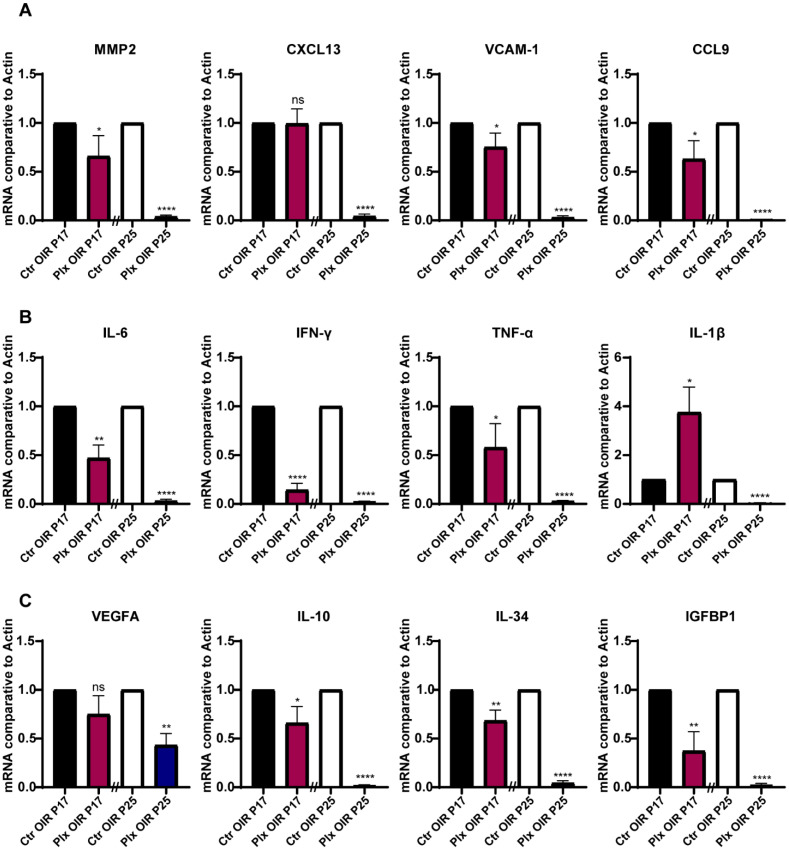
Expression of cytokines in the OIR and PLX groups on P17 and P25. (**A**–**C**) mRNA expression of the cytokines associated with adhesion (MMP2, CXCL13, VCAM-1, and CCL9), inflammation (IL-6, IFN-γ, TNF-α, and IL-1β), and angiogenesis (VEGFA, IL-10. IL-34, and IGFBP1) were determined by qRT-PCR (n = 3 per group). Bars = means ± SD; * *p* < 0.05; ** *p* < 0.01; **** *p* < 0.0001; NS, no significance.

For the purpose of exploring whether microglia regulated vascular growth through paracrine, we focused on the changes in the cytokine expression profiles in the retina during neovascularization after microglia removal on P17 and P25. Detecting the mRNA level by RT-qPCR, we found that the expressions of chemokines such as MMP2, VCAM-1, and CCL9 were significantly downregulated in the absence of microglia, while the expression of CXCL13 did not change obviously on P17 (Figure 4A). Next, we focused on the inflammatory cytokines, IL-6, IFN-γ, and TNF-α. All these pro-inflammatory cytokines showed significant downregulation except IL1-β, which was upregulated (Figure 4B). In terms of pro-angiogenic cytokines, the expression of IL-10, IL-34, and IGFBP1 were significantly downregulated after microglia were removed. The expression of VEGFA was downgraded as well, but there was no statistical difference (Figure 4C). The declined tendency of these cytokines could be observed on P25 after the depletion of microglia and was even more obvious on P25 (Figure 4A–C). In general, the chemokines, inflammatory factors, and pro-angiogenic factors in the retina were downregulated after the depletion of microglia during the whole pathological process, which also corresponded to the fact that PLX5622 had no apparent selectivity for the depletion of different subtypes of microglia. The results partially explained the phenomenon of reduced retinal angiogenesis after the depletion of microglia as well.

### 3.5. Microglia Played a Vital Role in Promoting Apoptosis in the Regression of OIR

**Figure 5:**
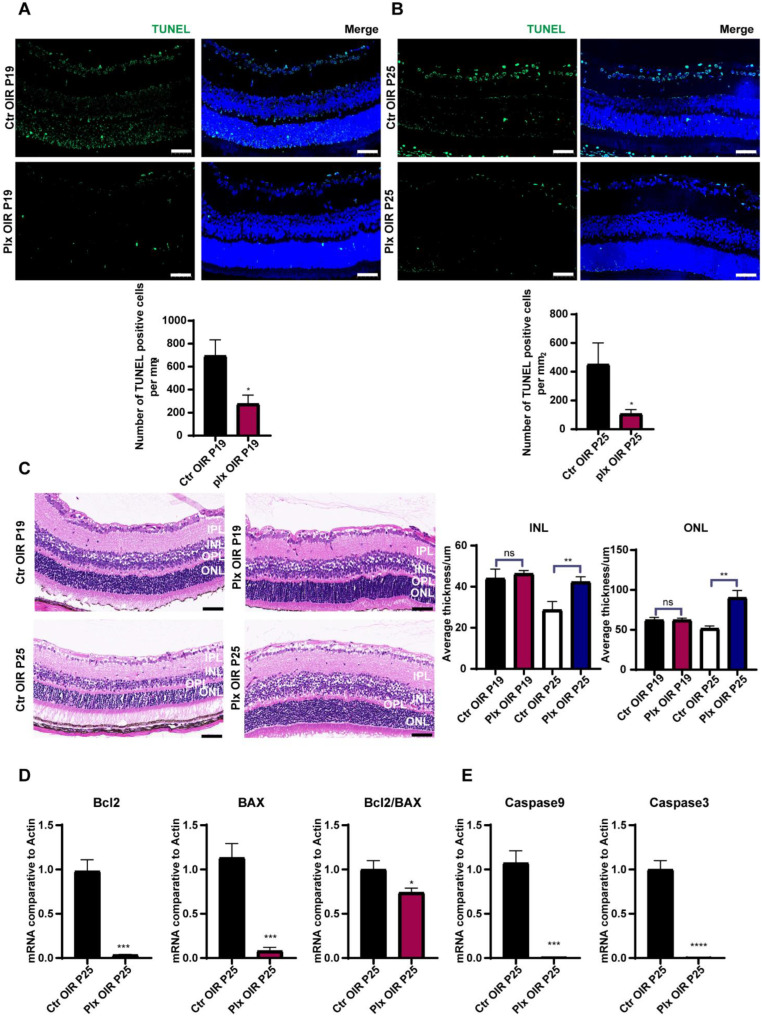
The apoptosis declined after microglial depletion on P25. (**A**,**B**) Immunofluorescence staining of TUNEL (green) and DAPI (blue) on P25 in both the Ctr OIR and PLX OIR groups (n = 3 per group). Scale bar, 100 µm. (**C**) HE staining of retinas in the Ctr OIR and PLX OIR groups (n = 3 per group). Scale bar, 100 µm. (**D**,**E**) mRNA expression of apoptosis-associated genes were determined by qRT-PCR (n = 3 per group). Bars = means ± SD; * *p* < 0.05; ** *p* < 0.01; *** *p* < 0.001; **** *p* < 0.0001; NS, no significance.

To find out the positive effects of microglia on visual function during vascular remodeling, combined with the previous reports on the microglia of pruning synapses and promoting neuronal apoptosis [31,32,33,34], we paid attention to the apoptosis of photoreceptor cells in the retina on P19 and P25. By TUNEL staining, we figured out that the PLX group showed a lower rate of apoptosis of retinal cells (mainly on photoreceptor cells and ganglion cells) on both P19 and P25 (Figure 5A,B). HE staining showed that there was no significant difference in the thickness of the inner and outer nuclear layers of the retina on P19. However, on P25, after the completion of vascular remodeling, the inner and outer nuclear layers in the PLX group were significantly thicker than those in the control group (Figure 5C). To find out the level of apoptosis in the retina, RT-qPCR was used to test the expression of apoptosis-associated genes, including Bcl2, Bax, Casp9, and Casp3 (Figure 5D,E). The ratio of Bcl2/BAX was decreased, and the expressions of Casp9 and Casp3 were downregulated significantly, which confirmed that the apoptosis was weakened after the depletion of microglia. Moreover, we compared the differences in the apoptosis of retinal cells by the IF staining of caspase 3 on P25 (Figure S3). It was also observed that the apoptosis declined in the photoreceptor cells. Taken together, our observation suggested that the primary function of microglia during this period was to promote the apoptosis of photoreceptor cells during retinal vascular remodeling. It might be achieved directly by regulating photoreceptor cells or indirectly by trimming the vascular network in the retina and reducing the energy supply of photoreceptor cells.

### 3.6. Depletion of Retinal Microglia Affected the Mobilization of Bone Marrow Cells

**Figure 6:**
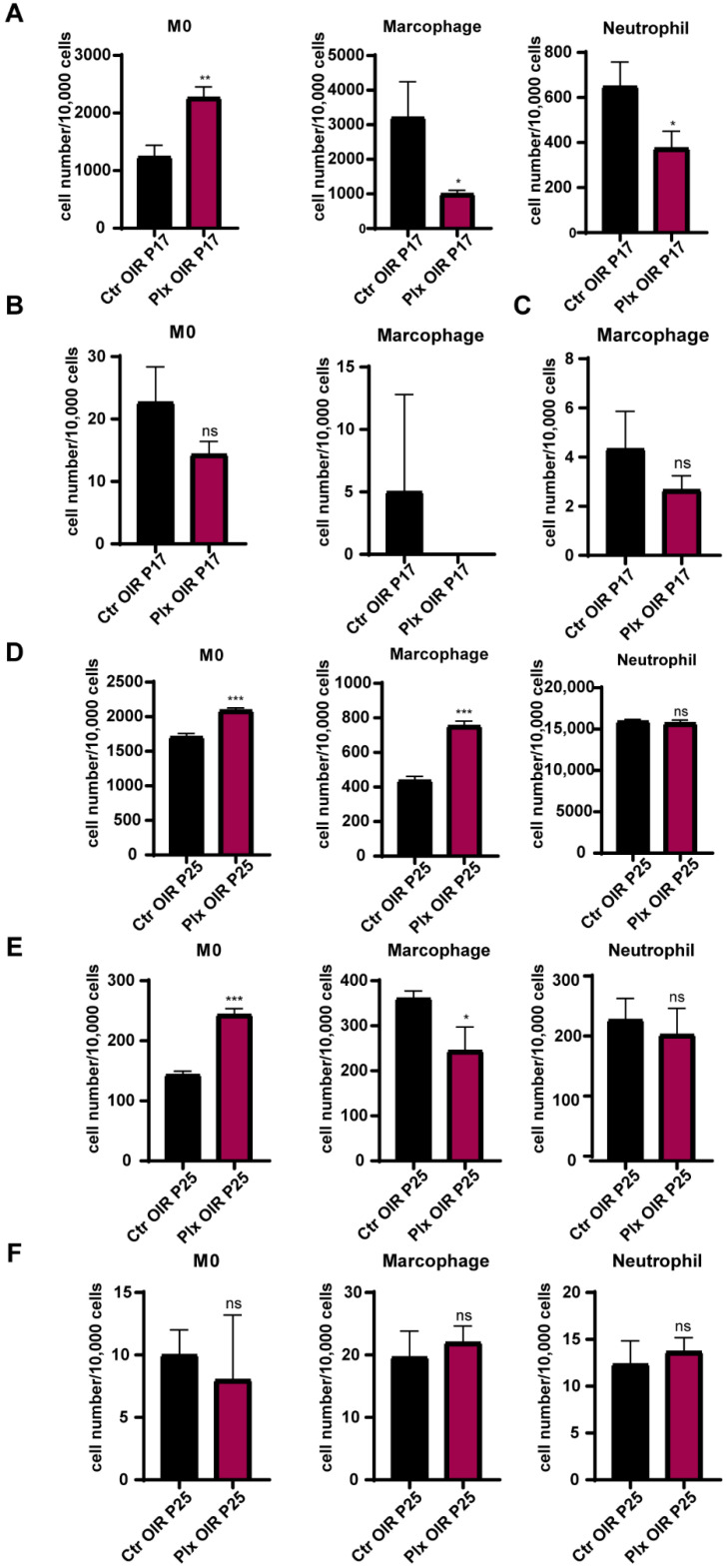
The changes in the distribution of immune cells after microglial depletion. The numbers of monocytes, macrophages, and neutrophils in bone marrow (**A**), peripheral blood (**B**), and retina (**C**) on P17 were quantified by FACs (n = 3 per group). The numbers of monocytes, macrophages, and neutrophils in bone marrow (**D**), peripheral blood (**E**), and retina (**F**) on P25 were quantified by FACs (n = 3 per group). Bars = means ± SD; * *p* < 0.05; ** *p* < 0.01; *** *p* < 0.001; NS, no significance.

To explore the effects of PLX5622 gavage on the mobilization of bone marrow cells and the role myeloid cells played in the changes above, flow cytometry was used to analyze the cells from bone marrow, peripheral blood, and retina of the OIR and PLX mice on P17 and P25, respectively. The results showed that on P17, the number of CD45 + CD11b + LY6Chigh monocytes increased significantly in bone marrow, while the number of CD45 + CD11b + LY6G + neutrophils and CD45 + CD11b + F4/80 + macrophages produced by bone marrow decreased significantly (Figure 6A). The specific gate in anchoring myeloid cells is shown in Figure S4. In the peripheral blood (Figure 6B) and retina (Figure 6C), the number of monocytes tended to decrease, while the number of neutrophils and macrophages could be ignored on P17, which was consistent with the previous study [35]. The neutrophils were not detected in peripheral blood, while M0 and neutrophils were not detected in retina in both the Ctr and PLX groups on P17. It might be because the immune cells had not yet fully distributed to the whole body. On P25, monocytes and macrophages in bone marrow increased significantly after microglial depletion, and the change of neutrophils was not significant (Figure 6D). Monocytes in blood also increased obviously, which might be caused by the migration of the increased number of monocytes in bone marrow to the peripheral blood. Meanwhile, macrophages and neutrophils in peripheral blood had a tendency to decrease, but there was no significant difference (Figure 6E). There was also no obvious difference in the number of myeloid cells in the retina of mice on P25 (Figure 6F). Previous studies [35,36] have shown that the pathological process of the OIR model did not affect the turnover rate of myeloid cells in the retina [35]. Our study showed that removing microglia by PLX5622 did not significantly affect the recruitment of myeloid cells in the retina, but it interfered with the proportion of myeloid cells in the bone marrow. The change could be manifested in the peripheral blood after a period of accumulation.

## 4. Discussion

Microglia were proven to take part in the vascular development of the retina [8]. By secreting pro- and anti-inflammatory cytokines, it reversed the cellular microenvironment after ischemic insult [13,14]. Intriguingly, we found that during the angiogenic stage of the OIR, both the neovascular area and vaso-occlusion area decreased after microglial removal. Moreover, the visual function of the PLX group was considerably worse than that of the control OIR mice. The results indicated the driving effects of microglia on neovascularization. By contrast, in the vascular regression stage of OIR, the vascular density, visual function, and the thickness of inner and outer nuclear layers of the retina in the PLX group dramatically increased. In order to find out an adequate explanation about this unexpected observation, we initially focused on the expression of cytokines in the retina after microglial removal. The results revealed that the expression of cytokines relating to adhesion, inflammation, and angiogenesis were downregulated in both P17 and 25. It might explain the decrease in neovascular area on P17, but could not fully account for different visual function between P17 and P25. As reported before, inhibition of microglia by CSF1R knock down led to decreased developmental apoptosis and increased density of retinal ganglion cells [37]. We turned our focus to retinal apoptosis. The apparent decrease in apoptosis of photoreceptor cells on P25 after microglial removal suggested that the role of microglia during the regression of neovascularization was different from that of the driving angiogenesis. Microglia might regulate photoreceptor cells by promoting their apoptosis directly, or they might reduce blood supply by pruning blood vessels, resulting in the death of photoreceptor cells indirectly, which still requires further studies.

In this study, we eliminated microglia in the retina of OIR mice by intragastric administration of the CSF1R inhibitor PLX5622 for the first time. PLX5622 is a targeted inhibitor of CSF1R, inhibiting the development, maintenance, and activation of microglia [38,39]. Studies have shown that when fed with CSF-1R inhibitors for one week, microglia in the retina were eliminated by up to 97% [26,27,40]. As reported, the reduction of antigen-presenting cells (APC) in the blood could be observed in the peripheral blood [41]. In our study, the CSF1R inhibitor had no lethal effect on immature mice and had no significant impact on the weight growth of mice, but it had a substantial effect on the proportion of monocytes and macrophages in the bone marrow, consistent with a previous study [42,43]. After inhibiting microglia by gene knockout, minocycline, and other methods, changes in cytokines could also be observed, and the changes in some cytokines and the degeneration of retinal cells were consistent with what we observed. At the same time, the combined use of minocycline and PLX5622 at the same time did not show additional changes [44,45]. In these circumstances, we believe that the changes in cytokines and apoptosis were caused by the removal of microglia instead of the drug itself.

As exposed in a previous study, the OIR model had few effects on the turnover rate of peripheral blood monocytes [35]. By comparing the composition of myeloid cells in the bone marrow, peripheral blood, and retina of mice, we found that the portion of myeloid cells in bone marrow and peripheral blood changed after microglial removal, while the difference of myeloid cells in the retina was minimal, which could be ignored. Collectively, after the depletion of microglia in the retina, few myeloid immune cells were recruited into the retina, indicating that microglial removal accounted for the changes rather than myeloid recruitment.

In these two stages, although microglia played different roles, it could be confirmed that microglia were amenable to promoting the pathological progression in both hypoxia-induced inflammation and ischemia models. Studies have shown that microglia participate in the development of choroidal neovascularization (CNV). Reducing the reactivity of immune cells through intravitreal injection of polysialic acid resulted in reduced vascular leakage in the laser-induced CNV model [46,47,48]. After microglia were removed with PLX5622, the NV area decreased significantly as well [49]. These studies suggested that the over-activation of microglia promoted the pathological progression. A large number of studies have shown that inhibiting the over-activation of immune cells might decelerate or alleviate the progress of the diseases. In a model of neuroinflammation induced by the West Nile virus encephalitis, PLX5622 inhibited the proliferation and lethal recruitment of immune cells into the infected brain, reducing neuroinflammation, and proved that PLX5622 was a potential target in immune cell-mediated diseases [43]. In our study, the inhibition of activated microglia reduced pathological angiogenesis in the early stage and then rescued the over-apoptosis of photoreceptors. Moreover, the therapy of PLX5622-mediated modulation of GAMM might be a novel therapy against glioma [50]. Therefore, local inhibition of microglia activation has considerable therapeutic prospects in ocular diseases. Thus, applying anti-VEGF drugs in combination with microglia inhibitors may bring a new dawn to patients with ocular neovascular diseases.

In summary, we removed the retinal microglia in natal OIR mice by gavage administration for the first time, which laid a solid foundation for the study of the exact role microglia played in ocular angiogenesis. Thus, we explored the distinct roles of microglia in different stages of the ocular neovascular disease. The in-depth study of the regulatory role of microglia in the pathological mechanisms suggested a better option for the treatment of ocular neovascular diseases.

## Figures and Tables

**Table 1:** Antibodies.

Name	Catalog	Purpose
CD31	BioLegend Cat# 102502	IF
α-SMA	Abcam ab184705	IF
CD45	BioLegend Cat# 147716	FACS
CD11b	BioLegend Cat# 101211	FACS
Ly6C	BioLegend Cat# 128022	FACS
Ly6G	BioLegend Cat# 127608	FACS
F4/80	BioLegend Cat# 123120	FACS
Rhodopsin	Cell Signaling Cat# 21782	IF
NeuN	Cell Signaling Cat# 24307	IF
Glast	Proteintech Cat#20785-1-AP	IF
Caspase3	Cell Signaling Cat# 9668	IF

**Table 2:** Primers.

Name	Forward (5′-3′)	Reverse (5′-3′)
Tmem119	AGGAGTCCTGAGAGATTTGCGC	GTGGATTCGGACCAGTCTGA
CD86	GTGGATTCGGAATTCAAGCTC	CAGCCAGTCTGAAGGCACA
iNOS	CATGATCGATCTTCGATATCGACG	TGACTGTAGCTAGCTATGCA
CD163	GTACGTACCATCGATGCATGCAT	TGATCTGATGCATGCTAGTACG
MMP2	CAAGGATGGACTCCTGGCACAT	CAAGGATGGACTCCTGGCACAT
CXCL13	CATAGATCGGATTCAAGTTACGCC	GTAACCATTTGGCACGAGGATTC
VCAM-1	GCTATGAGGATGGAAGACTCTGG	ACTTGTGCAGCCACCTGAGATC
CCL9	TCCAGAGCAGTCTGAAGGCACA	CCGTGAGTTATAGGACAGGCAG
IL-6	TACCACTTCACAAGTCGGAGGC	CTGCAAGTGCATCATCGTTGTTC
IFN-γ	CAGCAACAGCAAGGCGAAAAAGG	TTTCCGCTTCCTGAGGCTGGAT
TNF-α	GGTGCCTATGTCTCAGCCTCTT	GCCATAGAACTGATGAGAGGGAG
IL-1β	TGGACCTTCCAGGATGAGGACA	GTTCATCTCGGAGCCTGTAGTG
VEGFA	CTCTGGAACCTGAGACATCACC	AGGAGTCCTGAGAGATTTGCGC
Il-10	CGGGAAGACAATAACTGCACCC	CGGTTAGCAGTATGTTGTCCAGC
Il-34	ATCATCGGCATTTTGAACGAGGTC	ACCTTGGAAGCCCTACAGACGA
IGFBP1	GCCCAACAGAAAGCAGGAGATG	GTAGACACACCAGCAGAGTCCA
Bcl2	TGCATGTAACTACAGTACGACGTA	TGCATGACTCGTAGTCGATGCA
BAX	TGCATGCATAGCTAGTCGATGTCAT	TGCATATCGTAGCTGACAGCTG
Caspase3	GGAGTCTGACTGGAAAGCCGAA	CTTCTGGCAAGCCATCTCCTCA
Caspase9	GCTGTGTCAAGTTTGCCTACCC	CCAGAATGCCATCCAAGGTCTC
β-actin	GGCTGTATTCCCCTCCATCG	CCAGTTGGTAACAATGCCATG

## Data Availability

Not applicable.

## Supplementary Materials

The following supporting information can be downloaded at: https://www.mdpi.com/article/10.3390/life12101676/s1, Figure S1: The changes in vasculature during the two stages of OIR; Figure S2: Morphological and quantities changes in other retinal cells; Figure S3: The apoptosis of retinal cells; Figure S4: Flow cytometry analysis of myeloid population in the bone marrow from PLX5622 mice on P25.
