# Supplementary material for: Distinguished Functions of Microglia in the Two Stages of Oxygen-Induced Retinopathy: A Novel Target in the Treatment of Ischemic Retinopathy
Source: Life (Basel). 2022 Oct 21;12(10):1676. doi: 10.3390/life12101676 (PMC9604577; doi:10.3390/life12101676)
Supplement: Supplementary file 1 [file life-12-01676-s001.zip › life-1973094-supplementary.pdf]

Figure S1

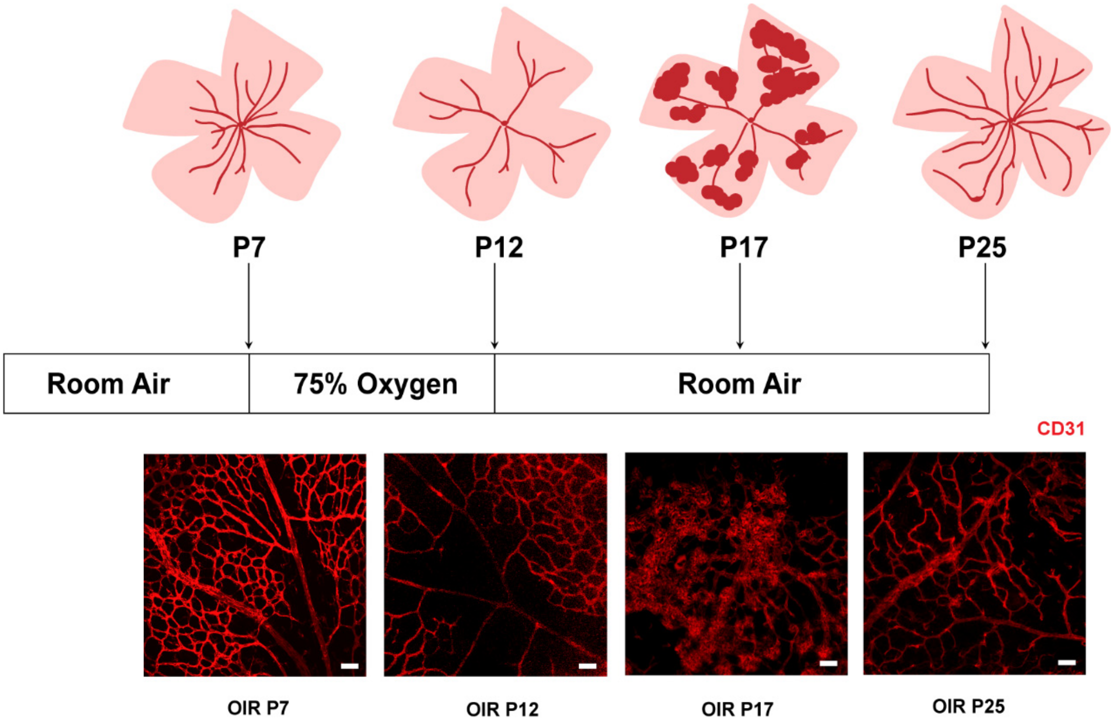

Figure S1 The changes in vasculature during the two stages of OIR.

Natal mice were placed in an oxygen box with 75% (±2%) from P7 to P12 to establish the OIR model, then were switched to room air at P12. The retinal vasculature degenerates during P7 to P12. The NV is formed during P12 to P17 and peaks on P17. The pathological NV regression happens after P17. The endothelial cells were stained by CD31. (n=3 per group). Scale bar, 100μm.

Figure S2

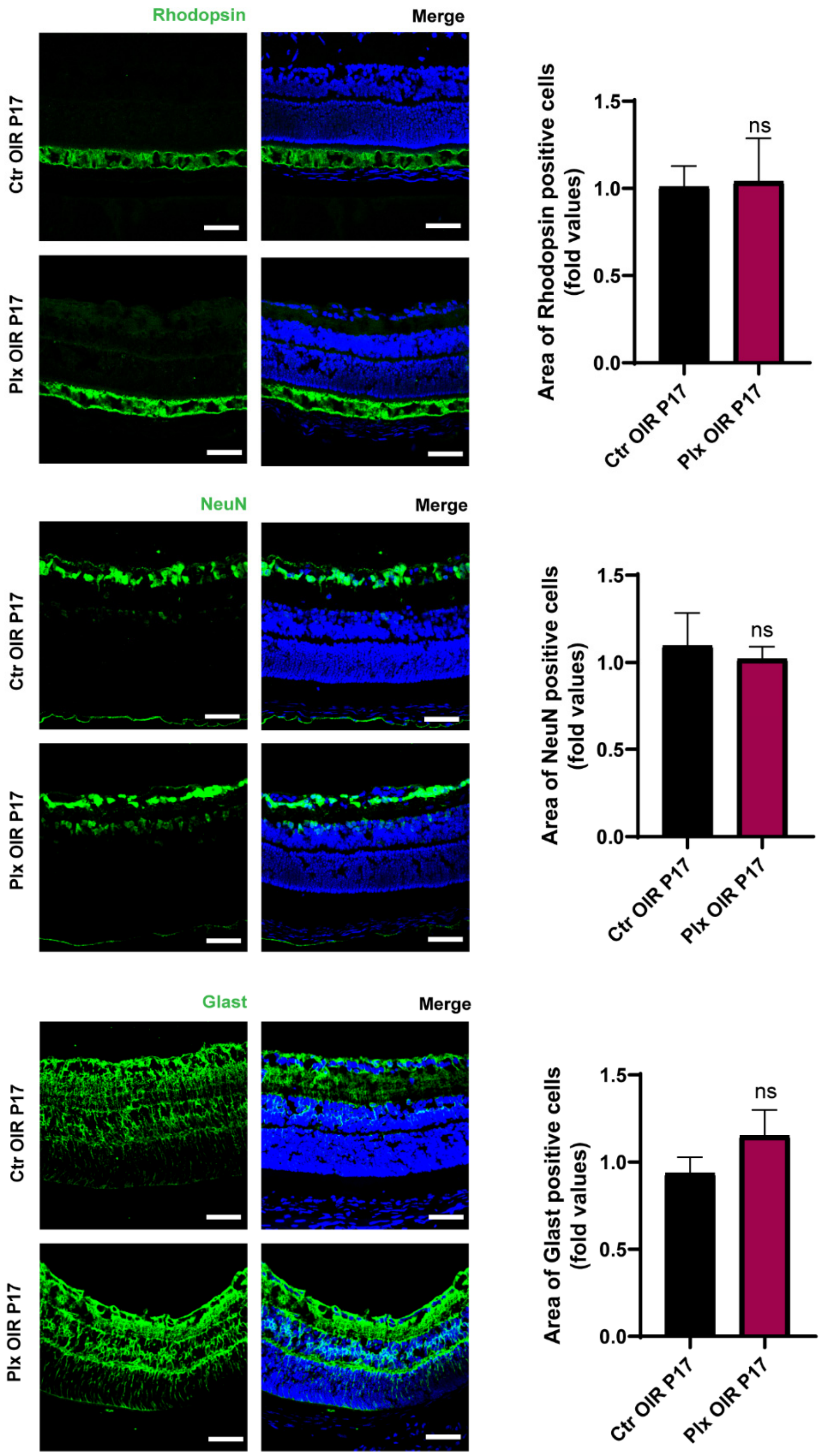

Figure S2 Morphological and quantities changes in other retinal cells.

Immunofluorescence staining of outer segment of photoreceptors (Rhodopsin, green), retinal ganglion cells (NeuN, green), macroglia (Glast, green), and DAPI

(blue) on P17 in both Ctr OIR and Plx OIR groups. Scale bar, 100μm (n=3 per group). Bars = means ± SD; NS, no significance.

Figure S3

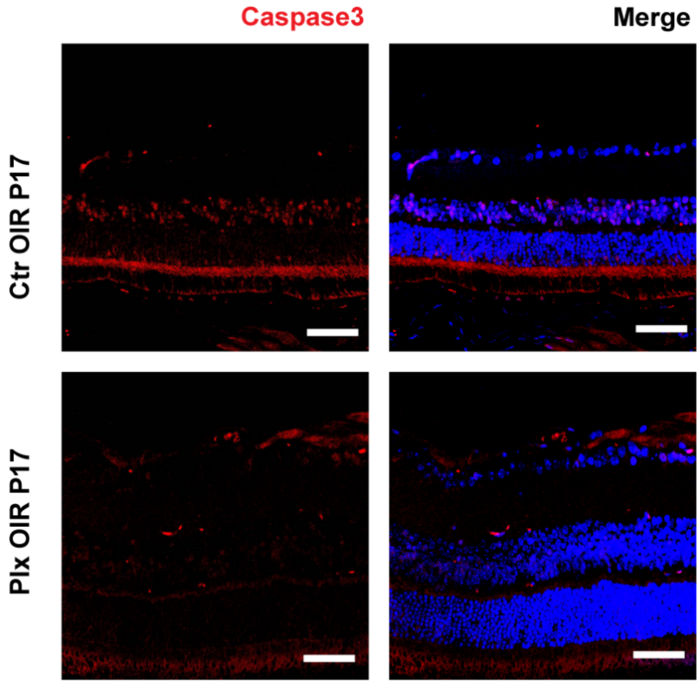

Figure S3 The apoptosis of retinal cells

Immunofluorescence staining of apoptotic cells (Caspase 3, red) and DAPI (blue) on P25 in both Ctr OIR and Plx OIR groups. Scale bar, 100μm (n=3 per group)

Figure S4

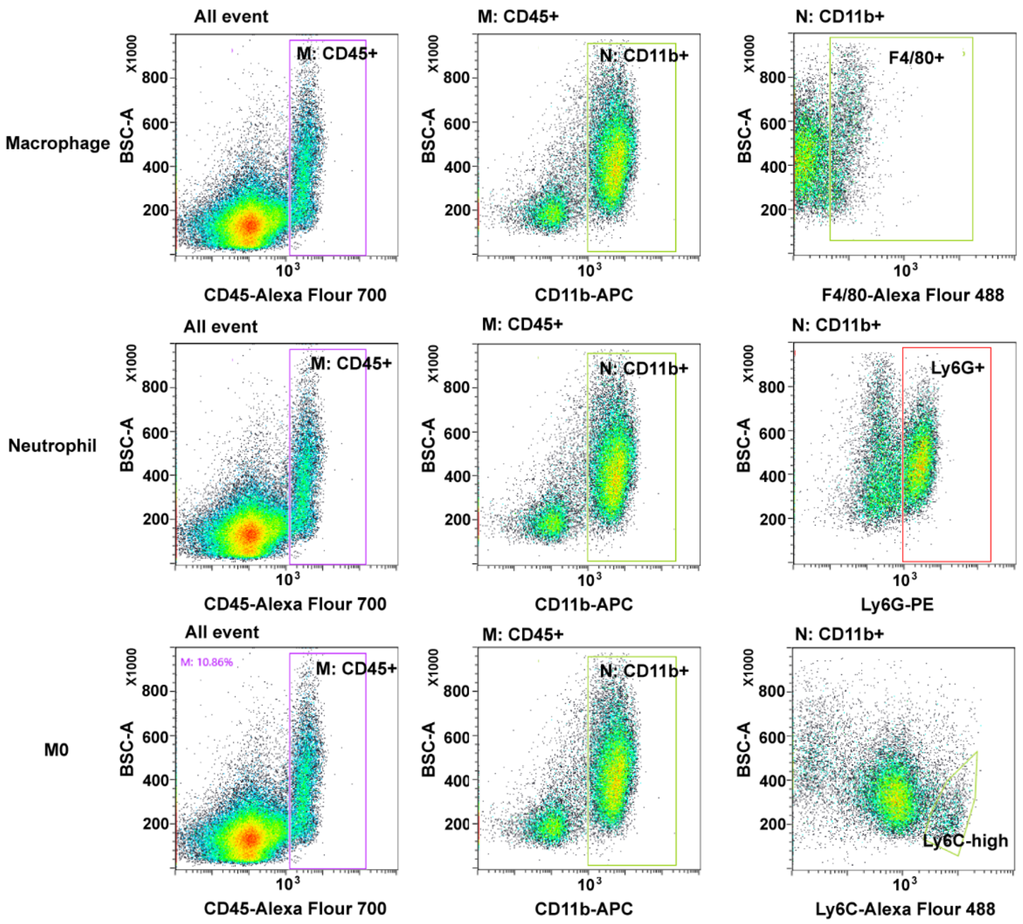

Figure S4 Flow cytometry analysis of myeloid population in the bone marrow from PLX5622 mice on P25

Representative flow cytometry plots of bone marrow from PLX5622 mice on P25. Macrophages were identified as CD45+CD11b+F4/80+. Neutrophils were identified as CD45+CD11b+LY6G+. Monocytes (M0) were identified as CD45+CD11b+LY6Chigh.
